# AAV9-Retro mediates efficient transduction with axon terminal absorption and blood–brain barrier transportation

**DOI:** 10.1186/s13041-020-00679-1

**Published:** 2020-10-14

**Authors:** Kunzhang Lin, Xin Zhong, Lei Li, Min Ying, Tian Yang, Zhijian Zhang, Xiaobin He, Fuqiang Xu

**Affiliations:** 1 Wuhan National Laboratory for Optoelectronics, Huazhong University of Science and Technology, Wuhan, 430074 China; 2 Center for Brain Science, State Key Laboratory of Magnetic Resonance and Atomic and Molecular Physics, Key Laboratory of Magnetic Resonance in Biological Systems, Wuhan Center for Magnetic Resonance, Wuhan Institute of Physics and Mathematics, Innovation Academy for Precision Measurement Science and Technology, Chinese Academy of Sciences, Wuhan, 430071 China; 3 University of Chinese Academy of Sciences, Beijing, 100049 PR China; 4 Shenzhen Key Lab of Neuropsychiatric Modulation, Guangdong Provincial Key Laboratory of Brain Connectome and Behavior, CAS Key Laboratory of Brain Connectome and Manipulation, The Brain Cognition and Brain Disease Institute (BCBDI), Shenzhen, Institutes of Advanced Technology, Chinese Academy of Sciences, Shenzhen-Hong Kong Institute of Brain Science-Shenzhen Fundamental Research Institutions, Shenzhen, 518055 China; 5 Center for Excellence in Brain Science and Intelligence Technology, Chinese Academy of Sciences, Shanghai, 200031 China

**Keywords:** Adeno-associated viruses, Retrogradely, AAV9-retro, Across the blood–brain barrier, Neural circuits

## Abstract

Recombinant adeno-associated viruses (rAAVs), particularly those that permit efficient gene transfer to neurons from axonal terminals or across the blood–brain barrier, are useful vehicles for structural and functional studies of the neural circuit and for the treatment of many gene-deficient brain diseases that need to compensate for the correct genes in every cell in the whole brain. However, AAVs with these two advantages have not been reported. Here, we describe a new capsid engineering method, which exploits the combination of different capsids and aims to yield a capsid that can provide more alternative routes of administration that are more suitable for the wide-scale transduction of the central nervous system (CNS). A new AAV variant, AAV9-Retro, was developed by inserting the 10-mer peptide fragment from AAV2-Retro into the capsid of AAV9, and the biodistribution properties were evaluated in mice. By intracranial and intravenous injection in the mice, we found that AAV9-Retro can retrogradely infect projection neurons with an efficiency comparable to that of AAV2-Retro and retains the characteristic of AAV9, which can be transported across the nervous system. Our strategy provides a new tool for the manipulation of neural circuits and future preclinical and clinical treatment of some neurological and neurodegenerative disorders.

## Introduction

Viral vectors, particularly those that permit efficient gene transfer to the central nervous system (CNS) from axonal terminals or across the blood–brain barrier, are useful to analyse the structure and function of specific neuronal circuits around the injection site [1–11] and have become one of the most promising therapy tools to deliver therapeutic genes to a distant target area [5, 12]. Compared with traditional retrograde tracers, viral vectors can express genes in specific neuron groups [9, 13] and have been widely used to monitor and manipulate neuronal activities by expressing optogenetic [14, 15], chemogenetic [16, 17] and calcium-sensitive functional probes [18–20]. Some natural and engineered neurotropic viruses exhibit retrograde infection capabilities, such as the pseudorabies virus (PRV) [21], herpes simplex virus (HSV) [22], rabies virus (RABV) [13, 23], lentivirus (LV) [24–27], canine adenovirus (CAV) [6, 28], and adeno-associated virus (AAV) [5, 29–31]. Among them, PRV is highly toxic [4, 32, 33]; HSV and RV can express genes rapidly and have high retrograde labelling efficiency, but they are also toxic to cells, limiting long-term gene manipulation [4, 13, 34, 35]. Rabies virus with double deletion of G/L has reduced the cytotoxicity and gene expression [35]. Lentiviruses packaged by modified RVG have high retrograde transport efficiency [25] but may induce host immune responses [36, 37] or lead to tumorigenesis by random insertion into the host genome [38]. CAV-2 has relatively low immunogenicity, large cloning capacity [39, 40], and enhanced retrograde labelling efficiency after compensation of the CAV-2 receptor in input neurons [6] but only moderate levels of gene expression [41] and difficulties in preparation [42].

Recombinant adeno-associated viruses (rAAVs) have become a powerful tool for neural circuit manipulation and gene therapy because they are low-toxic with high-level transgene expression and minimal host immune responses [43–45]. The treatment of many gene-deficient brain diseases must compensate for the correct genes to every cell in the whole brain [44]. Some natural and engineered adeno-associated viruses can transduce most CNS neurons by axon terminal absorption or blood–brain barrier transportation, such as AAV-TT [44], AAV2-Retro [5], AAV9-SLR [12], MNM004 [46], AAV-PHP.eB [47], and AAV-F [48], among others. However, AAVs with these two important features have not been reported. Compared with AAV2, AAV9 may have greater prospects for modification due to the advantages of lower neutralising antibodies in humans [49], higher transduction efficiency in vivo and the ability to cross the blood–brain barrier [50–52]. AAV9 can deliver therapeutic cargo throughout the CNS to partially correct phenotypes in mice models of CNS disease [53], including mucopolysaccharidosis (MPS) IIIB [54], amyotrophic lateral sclerosis (ALS) [55, 56], and Huntington’s disease [57], among others, and has been used to human clinical trials for the treament of spinal muscular atrophy (SMA, ClinicalTrials.gov: NCT02122952). AAV-TT exceeds the distribution abilities of AAV9 and AAVrh10 in the CNS of mice, and can correct the neurological phenotype in a mouse model of mucopolysaccharidosis IIIC [44]. Enhencing the distribution abilities (retrograde labeling) of AAV9 can provide more alternative routes of administration (intracranial or intravenous administration) that are more suitable for the wide-scale transduction of the central nervous system (CNS) and for gene therapy. Therefore, AAV9 variants that provide efficient transduction by axonal spread and across the blood–brain barrier are needed. Inserting the selected sequences from the AAV9 libraries into the capsid of AAV2 has been reported to not increase the transduction efficiency compared with AAV9 variants, and vice versa in most cases [58]. Therefore, it remains unknown whether the peptide segments from AAV2-Retro (AAV2 libraries) can be integrated into the capsid of AAV9 to achieve high-efficiency retrograde labelling and maintain the ability to cross the blood–brain barrier.

Herein, we inserted the 10-mer peptide fragment from AAV2-Retro (AAV2 libraries) into the capsid of AAV9 and compared it with AAV2-Retro in neurotropism. By intracranial and intravenous administration in mice, we found that this new variant, AAV9-Retro, can retrogradely infect projection neurons with efficiency comparable to AAV2-Retro and retain the ability to cross the blood–brain barrier. Our strategy provides a new retrograde tool for the manipulation of neural circuits and future treatment of some neurological and neurodegenerative diseases.

## Materials and methods

### Animals

Adult male (8–10 weeks old) C57BL/6J (Hunan SJA Laboratory Animal Company) mice were used for all experiments. The mice were housed in the appropriate environment with a 12/12-h light/dark cycle; water and food were supplied ad libitum*.* All the surgical and experimental procedures were performed in accordance with the guidelines formulated by the Animal Care and Use Committee of Wuhan Institute of Physics and Mathematics, Chinese Academy of Sciences.

### AAV9 capsid modification

The 10-mer peptide sequence (LADQDYTKTA) from AAV2-Retro (Addgene plasmid # 81070) was inserted between Q588 and A589 of the AAV9 capsid in the pAAV-RC9 vector (BrainVTA Technology Co., Ltd.). The corresponding 30-bp DNA sequence was introduced into the AAV9 capsid by overlap PCR and cloned into the pAAV-RC9 vector via the restriction enzymes BsiWI and NheI (New England Biolabs). The modified AAV9 capsid plasmids (AAV9-Retro) were verified by DNA sequencing.

### Recombinant AAV vector production

The various AAV serotype vectors, including AAV2-Retro-CaMKIIa-EGFP, AAV9-Retro-CaMKIIa-EGFP and AAV2-Retro-CaMKIIa-mCherry, were produced by a baculovirus-AAV expression vector system [59] and purified by iodixanol gradient ultracentrifugation [60, 61]. The purified rAAVs were titred by qPCR using the iQ SYBR Green Supermix kit (Bio-Rad) and diluted to 1.0 × 10^13^ viral particles/mL.

### Administration of AAV particles

The stereotactic injection coordinates were selected according to Paxinos and Franklin’s *The Mouse Brain in Stereotaxic Coordinates*, 4th edition [62]. The stereotactic coordinates for the VTA were as follows: AP: -3.20 mm; ML: ± 0.45 mm; DV: − 4.30 mm from the bregma. The stereotactic coordinates for CPu were as follows: AP: + 0.38 mm; ML: ± 2.00 mm; DV: − 3.50 mm from the bregma. Eight- to ten-week-old C57BL/6 J mice (20–25 g) were used for aav virus injection, and the standard injection process was performed as previously reported [11]. For single VTA site injection, the mice were divided into two groups (N = 3 in each group) and 300 nl of rAAV2-Retro-CaMKIIa-EGFP and rAAV9-Retro-CaMKIIa-EGFP viruses were infused into the VTA of each group. For mixed viral injection into the VTA, rAAV2-Retro-CaMKIIa-mCherry and rAAV9-Retro-CaMKIIa-EGFP viruses were mixed at the particle ratio of 1:1 and injected into the VTA at 300 nl. For single CPu site injection, the mice were divided into two groups (N = 3 in each group) and 300 nl of rAAV2-Retro-CaMKIIa-EGFP and rAAV9-Retro-CaMKIIa-EGFP viruses were infused into the CPu region of each group. After 3 weeks of virus expression in vivo, the mice were sacrificed using the conventional cardiac perfusion method. EGFP-expressing rAAV9, rAAV9-Retro and rAAV2-Retro (5 × 10^11^ vg/mouse) was intravenously injected into adult mice respectively, and the mice were sacrificed after 4 weeks of expression.

### Slice preparation and imaging

The brains were soaked with 4% paraformaldehyde solution overnight, dehydration was accomplished at 37 ℃ with 30% sucrose solution, and the brain was sectioned at a thickness of 40 μm using a frozen section instrument, and the brain slices were retrieved at 200-μm intervals. The brain slices were washed 3 times with PBS, 5–10 min each time. After DAPI staining (diluted by PBS at 1:3000) for 10–15 min, the brain slices were washed with PBS 2 times and applied neatly on the microscope slides, followed by sealing with 70% glycerol. Imaging was performed using the Leica TCS SP8 confocal microscope (Leica, Germany) or Olympus VS120 Slide Scanner microscope (Olympus, Japan).

### Statistical analysis

For cell counting, the images were divided into different brain regions using Adobe Photoshop CS5 according to the Allen Mouse Brain Atlas (https://www.brain-map.org/). The EGFP-positive neurons were quantified using ImageJ software, and the cells from the olfactory bulb were not counted. Data analysis was performed using unpaired t-test and GraphPad Prism (version 5.01; San Diego, CA). The data are shown as means ± SEM. The statistical significance of differences was set at P < 0.05. Graphs of virus injection were drawn using Sigma Plot (version 10.0; Systat Software Inc, San Jose, CA).

## Results

### Efficient retrograde connectivity tracing by the rAAV9-Retro variant

**Fig. 1:**
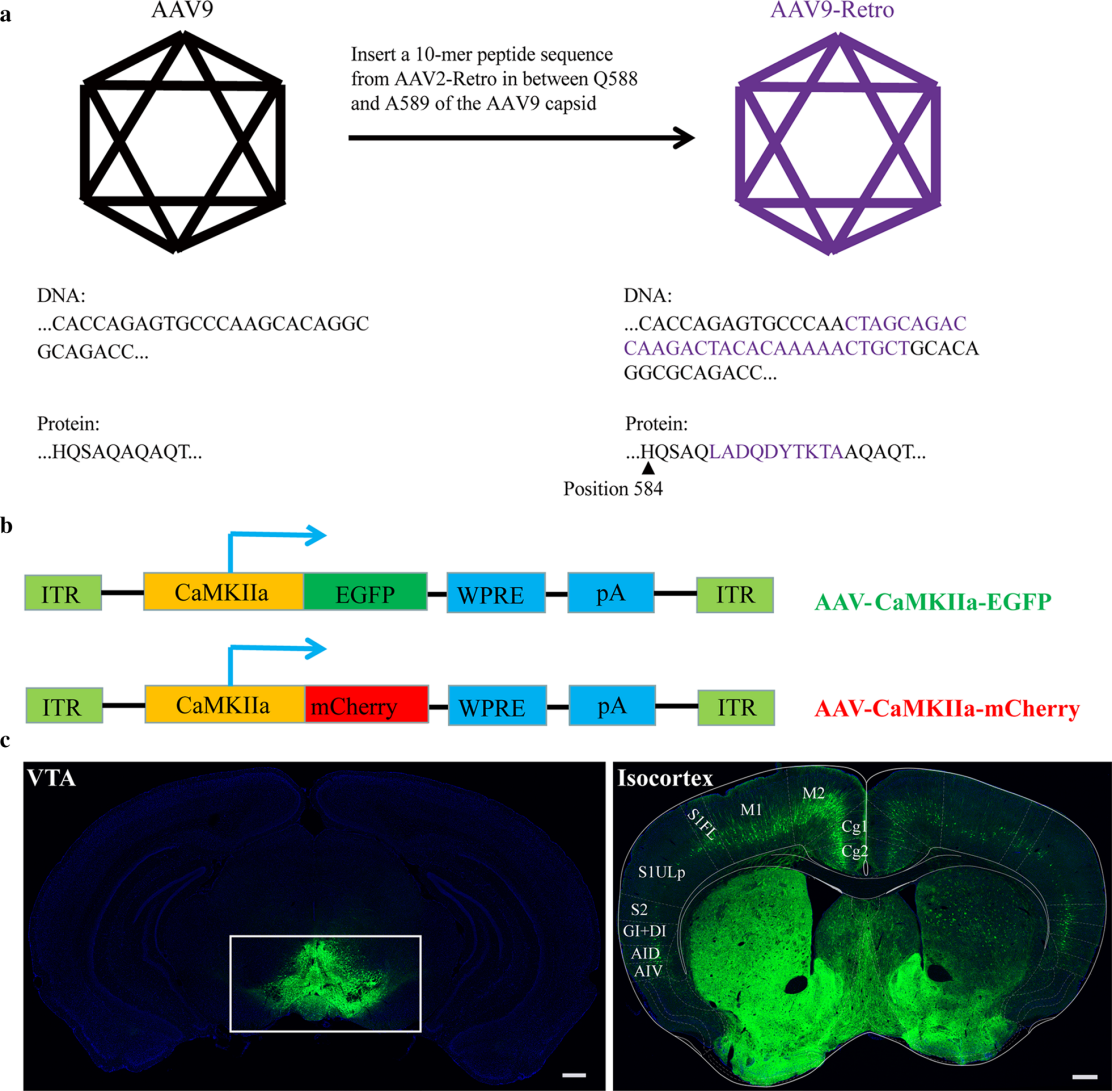
Efficient retrograde transduction by rAAV9-Retro. **a** The 10-mer peptide sequence (LADQDYTKTA) from AAV2-Retro (AAV2 libraries) was inserted between Q588 and A589 of the AAV9 capsid to produce AAV9-Retro. **b** The AAV-CaMKIIa-mCherry and AAV-CaMKIIa-EGFP vectors were used for packaging into viruses as a specific indicator of neurons. **c** Transduction validation of mouse brain neurons in vivo using AAV9-Retro. The AAV-CaMKIIa-EGFP vector was packaged into the AAV9-Retro virus, and then 300 nl of rAAV9-Retro-CaMKIIa-EGFP virus was infused into the VTA region, followed by local infection. The cortical region projecting to the VTA was imaged by confocal microscopy at 21 days post-injection (DPI). Many green fluorescent signals were found in the VTA and cortex. Scale bars = 1 mm. VTA: ventral tegmental area

Previous reports have shown that inserting the selected sequences from the AAV9 libraries into the capsid of AAV2 could not increase the transduction efficiency compared with the AAV9 variants [58], and the 7-mer PHP.B peptide (TLAVPFK) from the AAV9 libraries can improve the transduction efficiency of AAV1 in vivo and in vitro when inserted between S588 and T589 of the AAV1 capsid [63]. Therefore, whether the peptide segments from AAV2-Retro (AAV2 libraries) can be integrated into the capsid of AAV9 to achieve high-efficiency retrograde labelling remains to be verified. Thus, the 10-mer peptide sequence (LADQDYTKTA) from AAV2-Retro (AAV2 libraries) was inserted between Q588 and A589 of the AAV9 capsid to produce AAV9-Retro (Fig. 1a). rAAV-CaMKIIa-EGFP and rAAV-CaMKIIa-mCherry were packaged using AAV9-Retro (Fig. 1b). To evaluate whether AAV9-Retro can achieve high-efficiency retrograde labelling input neurons, 300 nl of the rAAV9-Retro-CaMKIIa-EGFP virus was infused into the VTA, and then local infection and the cortical region projecting to the VTA were imaged by confocal microscopy at 21 days post-injection (DPI). Many green fluorescent signals were found in the VTA and cortex (Fig. 1c), indicating that AAV9-Retro has high-efficiency retrograde access to input neurons.

### rAAV9-Retro shows a similar retrograde gene transduction efficiency to rAAV2-Retro

**Fig. 2:**
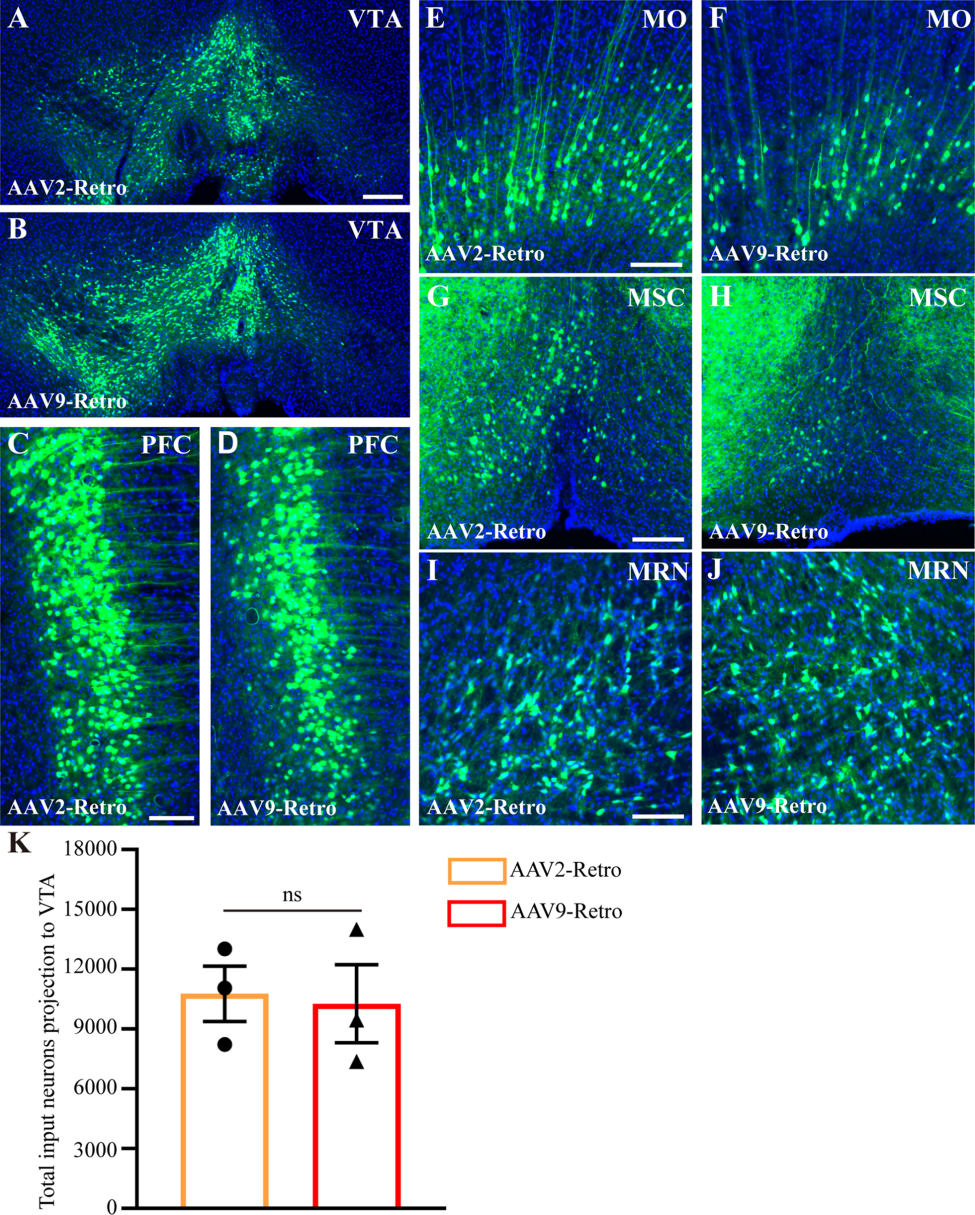
Comparison of the transduction efficiency between rAAV9-Retro and rAAV2-Retro injected into the VTA. **A**, **B** rAAV2-Retro-CaMKIIa-EGFP and rAAV9-Retro-CaMKIIa-EGFP viruses can efficiently infect neurons when injected into the VTA sites. **C**–**J** The two viruses can infect the same upstream brain regions, including the PFC, MO, MSC and MRN. **k** Comparison of the total number of neurons projecting into the VTA using rAAV9-Retro and rAAV2-Retro. The data are shown as means ± SEM. Statistical significance of differences was set at P < 0.05. No statistical difference was found in the total number of neurons projecting into the VTA (10,770 ± 1394 for AAV2-Retro; 10,278 ± 1957 for AAV9-Retro. P = 0.8477). Scale bars = 200 μm for Fig A-B; Scale bars = 100 μm for figure **C**–**J**. VTA: ventral tegmental area; PFC: prefrontal cortex; MO: somatomotor areas; MSC: medial septal complex; MRN: midbrain reticular nucleus

rAAV2-Retro has higher retrograde transduction efficiency than AAV9 and plays an important role in analysing nerve circuits [5]. To compare the retrograde infection efficiency of the rAAV9-Retro virus (based on AAV9 modification) with rAAV2-Retro, the two viruses were injected into the VTA sites (Fig. 2A, B). We found that the two viruses could infect the same upstream brain regions (Fig. 2C–J), including the prefrontal cortex (PFC), somatomotor areas (MO), medial septal complex (MSC) and midbrain reticular nucleus (MRN), and no statistical difference was found in the total number of neurons projected into the VTA (Fig. 2K, 10,770 ± 1394 for AAV2-Retro; 10,278 ± 1957 for AAV9-Retro; P = 0.8477). These results indicate that rAAV9-Retro and rAAV2-Retro have similar retrograde infection efficiency.

### rAAV9-Retro and rAAV2-Retro can retrogradely label the same projection neurons through mixed injection

**Fig. 3:**
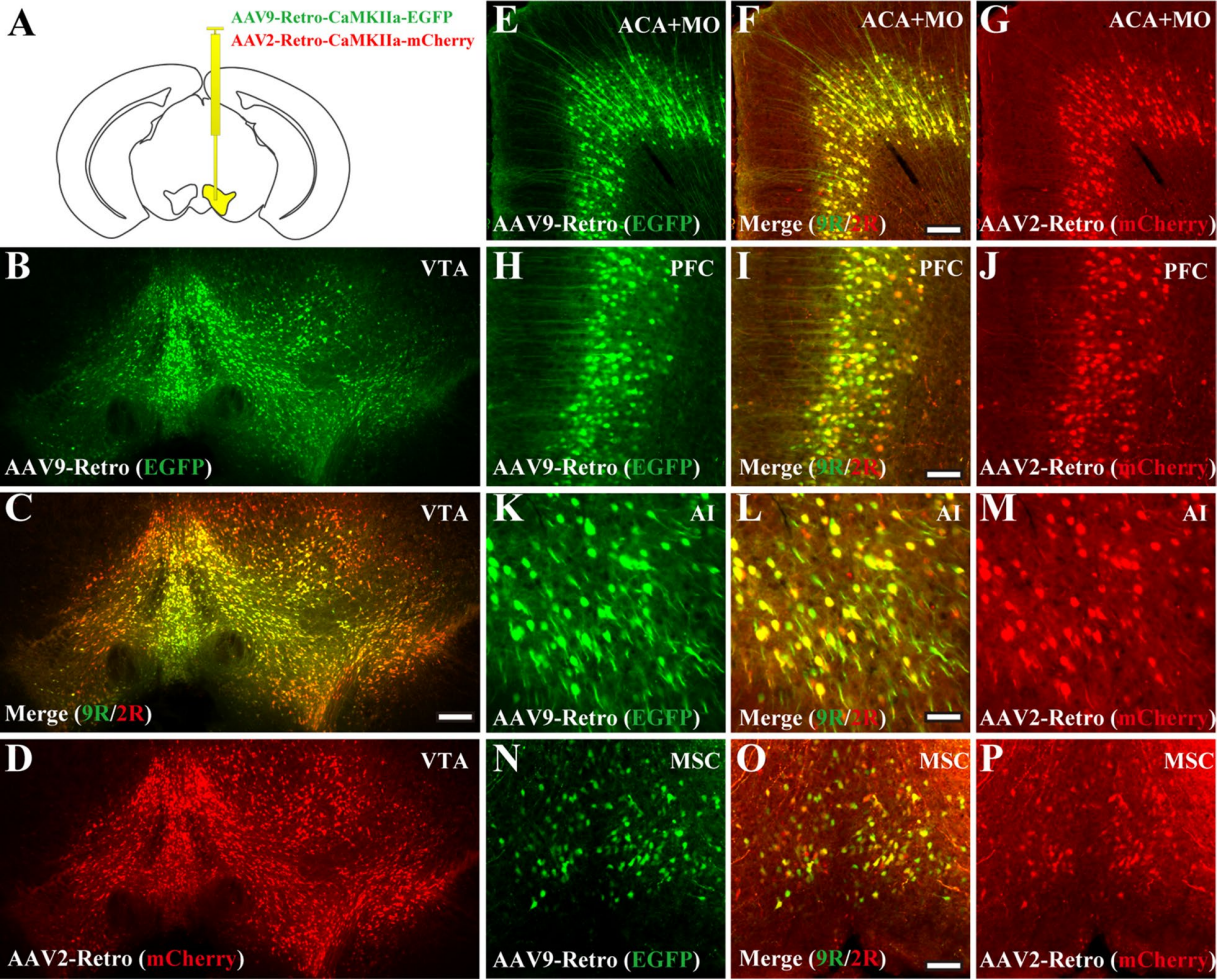
rAAV9-Retro and rAAV2-Retro can retrogradely label the same projection neurons through mixed injection. **a** rAAV2-Retro-CaMKIIa-mCherry and rAAV9-Retro-CaMKIIa-EGFP viruses were mixed at the particle ratio of 1:1 and were injected into VTA at 300 nl. Local infection and projection regions were imaged using the Olympus VS120 Slide Scanner microscope at 21 days post-injection (DPI). **B**–**P** Both green and red fluorescent signals appeared in VTA (**B**–**D**) and in the same brain areas with projection into the VTA (**E**–**P**), including ACA, MO, PFC, AI and MSC, and most of them were overlapped. Scale bars = 200 μm. ACA: anterior cingulate area; MO: somatomotor areas; PFC: prefrontal cortex; AI: agranular insular area; MSC: medial septal complex

To verify whether rAAV9-Retro and rAAV2-Retro can retrogradely infect the same brain regions that project to the injection site and avoid the difference caused by injection site deviation or individual variation of animals, rAAV2-Retro-CaMKIIa-mCherry and rAAV9-Retro-CaMKIIa-EGFP viruses were mixed at the particle ratio of 1:1 and injected into the VTA at 300 nl (Fig. 3A), and local infection and projection regions were imaged using an Olympus VS120 Slide Scanner microscope at 21 days post-injection (DPI). Both green and red fluorescent signals appeared in the VTA (Fig. 3B–D) and same brain areas that project into the VTA (Fig. 3E–P), such as the anterior cingulate area (ACA), somatomotor areas (MO), prefrontal cortex (PFC), agranular insular area (AI) and medial septal complex (MSC), with most of them overlapping. These results showed that rAAV9-Retro and rAAV2-Retro could retrogradely label the same projection neurons with mixed injection to the same brain area.

### rAAV9-Retro and rAAV2-Retro exhibit similar retrograde infection tropism and efficiency at different brain regions

**Fig. 4:**
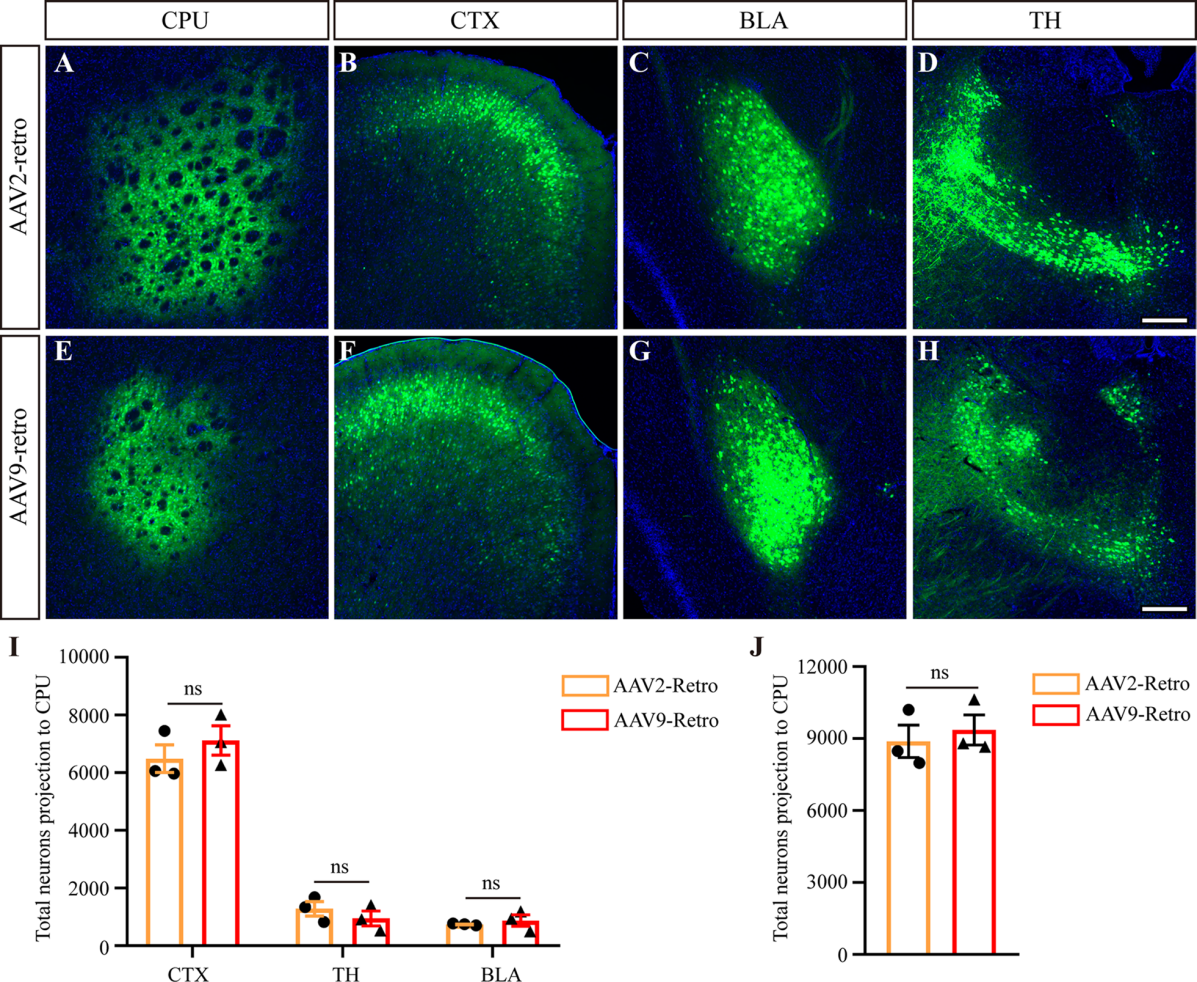
Comparison of retrograde infection tropism and efficiency between rAAV9-Retro and rAAV2-Retro at different brain regions. **A**–**H** The two viruses were injected into the brain area of CPu (**A**, **E**), which was reported to mainly receive input from the CTX, BLA and TH areas via rAAV2-Retro. Similar to rAAV2-Retro (**B**–**D**), rAAV9-Retro effectively infected these brain regions (**F**–**H**). **I**, **J** Through quantitative analysis, we found that the efficiency was equivalent between them (**i**, from CTX: 6495 ± 479.8 for AAV2-Retro, 7119 ± 507.6 for AAV9-Retro, P = 0.4219; from BLA: 737.3 ± 19.19 for AAV2-Retro, 871 ± 203.1 for AAV9-Retro, P = 0.5481; from TH: 1281 ± 250.0 for AAV2-Retro, 945.0 ± 256.6 for AAV9-Retro, P = 0.4010). Additionally, no statistical difference was found in the total number of neurons projected into the CPu (**J**, 8888 ± 672.8 for AAV2-Retro, 9366 ± 633.6 for AAV9-Retro; P = 0.6318). The data are shown as means ± SEM. Statistical significance of the differences was set at P < 0.05. Scale bars = 200 μm. CPu: caudate putamen (striatum); CTX: cerebral cortex; BLA: basolateral amygdalar nucleus; TH: thalamus

Viruses may have different infective characteristics and efficiency when injected into different brain areas. Therefore, we injected the two viruses into the brain area of the caudate putamen (CPu; Fig. 4A, E), which was reported to mainly receive input from the cerebral cortex (CTX), basolateral amygdalar nucleus (BLA) and thalamus (TH) via rAAV2-Retro [5]. We found that, like rAAV2-Retro (Fig. 4B–D), rAAV9-Retro effectively infected these brain regions (Fig. 4F–H), and the efficiency was equivalent between them through quantitative analysis (Fig. 4I, from CTX: 6495 ± 479.8 for AAV2-Retro, 7119 ± 507.6 for AAV9-Retro, P = 0.4219; from BLA: 737.3 ± 19.19 for AAV2-Retro, 871 ± 203.1 for AAV9-Retro, P = 0.5481; from TH: 1281 ± 250.0 for AAV2-Retro, 945.0 ± 256.6 for AAV9-Retro, P = 0.4010). Additionally, no statistical difference was found in the total number of neuronal projections to CPu (Fig. 4J; 8888 ± 672.8 for AAV2-Retro, 9366 ± 633.6 for AAV9-Retro; P = 0.6318). These results indicate that rAAV9-Retro and rAAV2-Retro have similar retrograde infection tropism and efficiency at different brain regions.

### rAAV9-Retro mediates efficient transduction across the central nervous systems after intravenous administration

**Fig. 5:**
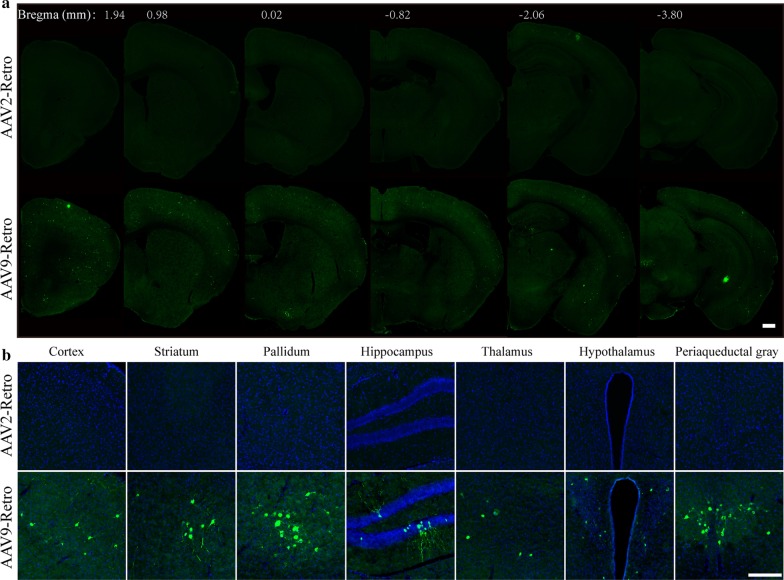
rAAV9-Retro can transduce neurons across multiple CNS regions after intravenous administration. EGFP-expressing rAAV9-Retro or rAAV2-Retro (5 × 10^11^ vg/mouse) was intravenously injected into adult mice. **a** Representative images of the EGFP signal in the brains of mice after 4 weeks of expression. **b** High magnification images of the brain sections shown in **a**. Scale bars = 500 μm for **a** and 200 μm for **b**

**Fig. 6:**
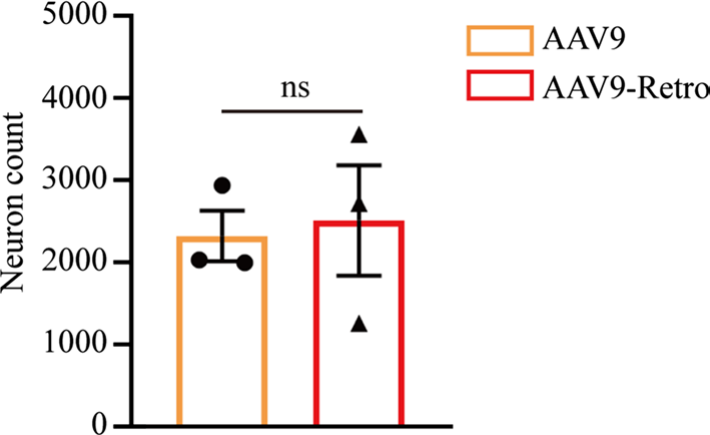
AAV9-Retro mediates efficient transduction across the CNS with an efficiency comparable to that of AAV9. EGFP-expressing rAAV9 or rAAV9-Retro (5 × 10^11^ vg/mouse) was intravenously injected into adult mice. No statistical difference was found in the total number of EGFP-positive neurons in the brain (2323 ± 308.6 for AAV9, 2513 ± 672.2 for AAV9-Retro; P = 0.8096). The data are shown as means ± SEM. Statistical significance of the differences was set at P < 0.05

Compared with AAV2, AAV9 has the advantage of transduction across the blood–brain barrier. To verify whether rAAV9-Retro can mediate efficient transduction across the central nervous systems, EGFP-expressing rAAV9、rAAV9-Retro and rAAV2-Retro (5 × 10^11^ vg/mouse) was intravenously injected into adult mice respectively, and the fluorescence signals in the brains of mice were imaged after 4 weeks of expression. We found that rAAV9-Retro allowed efficient transduction across the blood–brain barrier after intravenous administration (Fig. 5a), and EGFP signals were observed in several important brain regions (including the cortex, striatum, pallidum, hippocampus, thalamus, hypothalamus and periaqueductal grey), which were not shown in rAAV2-Retro (Fig. 5b). Importantly, no statistical difference between rAAV9 and rAAV9-Retro was found in the total number of EGFP-positive neurons in the brain (Fig. 6, 2323 ± 308.6 for AAV9, 2513 ± 672.2 for AAV9-Retro; P = 0.8096). These results indicate that AAV9-Retro can mediate efficient transduction across the CNS with an efficiency comparable to that of AAV9.

## Discussion

Adeno-associated viruses play an important role in understanding the structure and function of the brain [64]. They are also the primary vectors in the field of gene therapy and can be used in the treatment of various genetic defects, such as Parkinson’s disease [65], haemophilia [66, 67], and lysosomal disease [68]. Herein, we have endowed the AAV9 capsid with efficient retrograde transduction capacity in the mouse brain by inserting the 10-mer peptide sequence (LADQDYTKTA) from AAV2-Retro (AAV2 libraries) between Q588 and A589. The retrograde infection tropism and efficiency of rAAV9-Retro are similar to those of rAAV2-Retro in neural circuits. Importantly, rAAV9-Retro retains the ability to deliver genes across the blood–brain barrier.

Various neurotropic viruses, such as HSV1 [22], CAV2 [6], RABV [13], HiRet and NeuRet lentivirus [25], which have been widely used in the study of brain circuits, have the characteristics of retrograde access to specific or multiple types of neurons [69, 70]. However, high toxicity makes them unsuitable for long-term functional studies in model animals, further limiting their application in disease treatment [4]. Although nontoxic rabies virus has been developed, its expression ability is weaker and needs to expand the expression of foreign genes by carrying CRE and FLP recombinase combined with transgenic animals or AAV viruses [35]. Several AAV variants have been developed for more efficient transduction of the nervous system. For example, rAAV2-Retro allows for the efficient mapping, monitoring, and manipulation of projection neurons [5]. Since the retrograde infection tropism and efficiency of rAAV9-Retro are similar to those of rAAV2-Retro in neural circuits, rAAV9-Retro can also be used for the structural mapping and functional manipulation of input neurons. In addition, due to less toxicity and high-efficiency retrograde labelling, rAAV9-Retro can replace CAV2 in TRIO system (for tracing the relationship between input and output) and cell-type-specific TRIO system (cTRIO) [71], which have been developed to determine the input–output organization of neural circuits [71, 72]. AAV9 has lower neutralising antibodies in vivo, and the AAV9 variant can evade the neutralisation reaction more effectively than AAV2 or AAV2 variant in vitro [49, 58]. In addition, increasing the efficiency of AAV9 transduction and decreasing the administration dosage can reduce the immune response caused by a dose increase [73]. Moreover, compared with AAV2, AAV9 is less affected by the change in the extracellular matrix in the brain [12]. Therefore, AAV9-Retro may be a better vector for functional network manipulation and gene therapy.

Non-human primates are deal experimental animals for human disease modeling [74, 75], some recombinant adeno-associated viruses (rAAVs) can undergo retrograde transport in non-human primates, but the efficiency of retrograde transport is low, limiting the analysis of functional circuits and implications for disease modeling [76]. AAV2-Retro can mediate extensive retrograde transport in the rhesus macaque brain [77], but whether AAV9-Retro can achieve high-efficiency retrograde labelling in non-human primates brain is unknown. Therefore, it will be meaningful to compare the retrograde transduction efficiency between AAV9-Retro and AAV2-Retro in non-human primates, which may pave the path to gene therapy.

The treatment of many gene-deficient brain diseases, such as mucopolysaccharidosis IIIC, needs to compensate for the correct genes to every cell in the whole brain [44]. AAV, with effective retrograde infection or greater diffusion capacity, can achieve a wider range of drug delivery. AAV9 has a unique advantage over other serotype viruses—that is, it can transport across the blood–brain barrier and target the whole brain by intravenous injection. The retrograde AAV based on AAV9 modification can also mediate efficient gene delivery to the brain by intravenous administration and be combined with local administration to achieve a better therapeutic effect, which will be addressed in a future study.

In summary, we have proven that AAV9-Retro can mediate efficient transduction with axon terminal absorption and blood–brain barrier transportation, providing an additional AAV vector tool for neuroscience and gene therapy.

## Data Availability

The datasets used or analysed during the current study are available from the corresponding author on reasonable request.

## Abbreviations

rAAV: Recombinant adeno-associated virus
CNS: Central nervous system
PRV: Pseudorabies virus
HSV: Herpes simplex virus
RABV: Rabies virus
LV: Lentivirus
CAV: Canine adenovirus
VTA: Ventral tegmental area
PFC: Prefrontal cortex
MO: Somatomotor areas
MSC: medial septal complex
MRN: midbrain reticular nucleus
ACA: anterior cingulate area
AI: agranular insular area
CPu: caudate putamen (striatum)
CTX: cerebral cortex
BLA: basolateral amygdalar nucleus
TH: thalamus
DPI: days post-injection

## References

[1] Smith BN, Banfield BW, Smeraski CA, Wilcox CL, Dudek FE, Enquist LW (2000). Pseudorabies virus expressing enhanced green fluorescent protein: a tool for in vitro electrophysiological analysis of transsynaptically labeled neurons in identified central nervous system circuits. Proc Natl Acad Sci USA.

[2] Wickersham IR, Lyon DC, Barnard RJ, Mori T, Finke S, Conzelmann KK (2007). Monosynaptic restriction of transsynaptic tracing from single, genetically targeted neurons. Neuron.

[3] Rothermel M, Brunert D, Klupp BG, Luebbert M, Mettenleiter TC, Hatt H (2009). Advanced tracing tools: functional neuronal expression of virally encoded fluorescent calcium indicator proteins. J Neurovirol.

[4] Ugolini G (2010). Advances in viral transneuronal tracing. J Neurosci Methods.

[5] Tervo DG, Hwang BY, Viswanathan S, Gaj T, Lavzin M, Ritola KD (2016). A Designer AAV variant permits efficient retrograde access to projection neurons. Neuron.

[6] Li SJ, Vaughan A, Sturgill JF, Kepecs A (2018). A viral receptor complementation strategy to overcome CAV-2 tropism for efficient retrograde targeting of neurons. Neuron..

[7] Sheikh IS, Keefe KM, Sterling NA, Junker IP, Eneanya CI, Liu Y (2018). Retrogradely transportable lentivirus tracers for mapping spinal cord locomotor circuits. Front Neural Circuits.

[8] Wang Z, Maunze B, Wang Y, Tsoulfas P, Blackmore MG (2018). Global connectivity and function of descending spinal input revealed by 3D microscopy and retrograde transduction. J Neurosci.

[9] Chen SH, Haam J, Walker M, Scappini E, Naughton J, Martin NP (2019). Recombinant viral vectors as neuroscience tools. Curr Protoc Neurosci.

[10] Sun L, Tang Y, Yan K, Yu J, Zou Y, Xu W (2019). Differences in neurotropism and neurotoxicity among retrograde viral tracers. Mol Neurodegener.

[11] Zhu X, Lin K, Liu Q, Yue X, Mi H, Huang X (2020). Rabies virus pseudotyped with CVS-N2C glycoprotein as a powerful tool for retrograde neuronal network tracing. Neurosci Bull.

[12] Commisso B, Ding L, Varadi K, Gorges M, Bayer D, Boeckers TM (2018). Stage-dependent remodeling of projections to motor cortex in ALS mouse model revealed by a new variant retrograde-AAV9. Elife..

[13] Wickersham IR, Finke S, Conzelmann KK, Callaway EM (2007). Retrograde neuronal tracing with a deletion-mutant rabies virus. Nat Methods.

[14] Zhang F, Wang LP, Brauner M, Liewald JF, Kay K, Watzke N (2007). Multimodal fast optical interrogation of neural circuitry. Nature.

[15] Gradinaru V, Zhang F, Ramakrishnan C, Mattis J, Prakash R, Diester I (2010). Molecular and cellular approaches for diversifying and extending optogenetics. Cell.

[16] Krashes MJ, Koda S, Ye C, Rogan SC, Adarns AC, Cusher DS (2011). Rapid, reversible activation of AgRP neurons drives feeding behavior in mice. J Clin Investig.

[17] Zhang Z, Ferretti V, Guntan I, Moro A, Steinberg EA, Ye Z (2015). Neuronal ensembles sufficient for recovery sleep and the sedative actions of alpha2 adrenergic agonists. Nat Neurosci.

[18] Broussard GJ, Liang Y, Fridman M, Unger EK, Meng G, Xiao X (2018). In vivo measurement of afferent activity with axon-specific calcium imaging. Nat Neurosci.

[19] Yang Y, Liu N, He Y, Liu Y, Ge L, Zou L (2018). Improved calcium sensor GCaMP-X overcomes the calcium channel perturbations induced by the calmodulin in GCaMP. Nat Commun.

[20] Reardon TR, Murray AJ, Turi GF, Wirblich C, Croce KR, Schnell MJ (2016). Rabies virus CVS-N2c(DeltaG) strain enhances retrograde synaptic transfer and neuronal viability. Neuron.

[21] DeFalco J, Tomishima M, Liu HY, Zhao C, Cai XL, Marth JD (2001). Virus-assisted mapping of neural inputs to a feeding center in the hypothalamus. Science.

[22] Ugolini G, Kuypers H, Simmons A (1987). Retrograde trans-neuronal transfer of herpes-simplex virus type-1 (HSV 1) from motoneurons. Brain Res.

[23] Callaway EM, Luo L (2015). Monosynaptic circuit tracing with glycoprotein-deleted rabies viruses. J Neurosci.

[24] Kato S, Kobayashi K, Inoue K, Kuramochi M, Okada T, Yaginuma H (2011). A lentiviral strategy for highly efficient retrograde gene transfer by pseudotyping with fusion envelope glycoprotein. Hum Gene Ther.

[25] Kato S, Kobayashi K, Inoue K-I, Takada M, Kobayashi K (2013). Vectors for highly efficient and neuron-specific retrograde gene transfer for gene therapy of neurological diseases. Gene Therapy.

[26] Kobayashi K, Inoue KI, Tanabe S, Kato S, Takada M, Kobayashi K (2017). Pseudotyped lentiviral vectors for retrograde gene delivery into target brain regions. Front Neuroanat.

[27] Tanabe S, Inoue KI, Tsuge H, Uezono S, Nagaya K, Fujiwara M (2017). The use of an optimized chimeric envelope glycoprotein enhances the efficiency of retrograde gene transfer of a pseudotyped lentiviral vector in the primate brain. Neurosci Res.

[28] Soudais C, Laplace-Builhe C, Kissa K, Kremer EJ (2001). Preferential transduction of neurons by canine adenovirus vectors and their efficient retrograde transport in vivo. Faseb J..

[29] Low K, Aebischer P, Schneider BL (2013). Direct and retrograde transduction of nigral neurons with AAV6, 8, and 9 and intraneuronal persistence of viral particles. Hum Gene Ther.

[30] Salegio EA, Samaranch L, Kells AP, Mittermeyer G, San Sebastian W, Zhou S (2013). Axonal transport of adeno-associated viral vectors is serotype-dependent. Gene Ther.

[31] San Sebastian W, Samaranch L, Heller G, Kells AP, Bringas J, Pivirotto P (2013). Adeno-associated virus type 6 is retrogradely transported in the non-human primate brain. Gene Ther.

[32] Pomeranz LE, Reynolds AE, Hengartner CJ (2005). Molecular biology of pseudorabies virus: impact on neurovirology and veterinary medicine. Microbiol Mol Biol Rev.

[33] McCarthy KM, Tank DW, Enquist LW (2009). Pseudorabies virus infection alters neuronal activity and connectivity in vitro. PLoS Pathog.

[34] Ciabatti E, Gonzalez-Rueda A, Mariotti L, Morgese F, Tripodi M (2017). Life-long genetic and functional access to neural circuits using self-inactivating rabies virus. Cell..

[35] Chatterjee S, Sullivan HA, MacLennan BJ, Xu R, Hou Y, Lavin TK (2018). Nontoxic, double-deletion-mutant rabies viral vectors for retrograde targeting of projection neurons. Nat Neurosci.

[36] Follenzi A, Santambrogio L, Annoni A (2007). Immune responses to lentiviral vectors. Curr Gene Ther.

[37] Annoni A, Gregori S, Naldini L, Cantore A (2019). Modulation of immune responses in lentiviral vector-mediated gene transfer. Cellular Immunol..

[38] Themis M, Waddington SN, Schmidt M, von Kalle C, Wang Y, Al-Allaf F (2006). Oncogenesis following delivery of a nonprimate lentiviral gene therapy vector to fetal and neonatal mice (vol 12, pg 763, 2005). Mol Ther.

[39] Perreau M, Kremer EJ (2005). Frequency, proliferation, and activation of human memory T cells induced by a nonhuman adenovirus. J Virol.

[40] Soudais C, Skander N, Kremer EJ (2003). Long-term in vivo transduction of neurons throughout the rat central nervous system using novel helper-dependent CAV-2 vectors. Faseb J..

[41] Simao D, Pinto C, Fernandes P, Peddie CJ, Piersanti S, Collinson LM (2016). Evaluation of helper-dependent canine adenovirus vectors in a 3D human CNS model. Gene Ther.

[42] Kremer EJ, Boutin S, Chillon M, Danos O (2000). Canine adenovirus vectors: an alternative for adenovirus mediated gene transfer. J Virol.

[43] Ojala DS, Amara DP, Schaffer DV (2015). Adeno-associated virus vectors and neurological gene therapy. Neuroscientist.

[44] Tordo J, O'Leary C, Antunes A, Palomar N, Aldrin-Kirk P, Basche M (2018). A novel adeno-associated virus capsid with enhanced neurotropism corrects a lysosomal transmembrane enzyme deficiency. Brain.

[45] Hampson DR, Hooper AWM, Niibori Y (2019). The Application of Adeno-Associated Viral Vector Gene Therapy to the Treatment of Fragile X Syndrome. Brain Sci..

[46] Davidsson M, Wang G, Aldrin-Kirk P, Cardoso T, Nolbrant S, Hartnor M (2019). A systematic capsid evolution approach performed in vivo for the design of AAV vectors with tailored properties and tropism. Proc Natl Acad Sci USA.

[47] Chan KY, Jang MJ, Yoo BB, Greenbaum A, Ravi N, Wu W-L (2017). Engineered AAVs for efficient noninvasive gene delivery to the central and peripheral nervous systems. Nat Neurosci.

[48] Hanlon KS, Meltzer JC, Buzhdygan T, Cheng MJ, Sena-Esteves M, Bennett RE (2019). Selection of an efficient AAV vector for robust CNS transgene expression. Mol Ther Methods Clin Dev.

[49] Boutin S, Monteilhet V, Veron P, Leborgne C, Benveniste O, Montus MF (2010). Prevalence of serum IgG and neutralizing factors against adeno-associated virus (AAV) types 1, 2, 5, 6, 8, and 9 in the healthy population: implications for gene therapy using AAV vectors. Hum Gene Ther.

[50] Inagaki K, Fuess S, Storm TA, Gibson GA, McTiernan CF, Kay MA (2006). Robust systemic transduction with AAV9 vectors in mice: Efficient global cardiac gene transfer superior to that of AAV8. Mol Ther..

[51] Zincarelli C, Soltys S, Rengo G, Rabinowitz JE (2008). Analysis of AAV serotypes 1–9 mediated gene expression and tropism in mice after systemic injection. Mol Ther.

[52] Foust KD, Nurre E, Montgomery CL, Hernandez A, Chan CM, Kaspar BK (2009). Intravascular AAV9 preferentially targets neonatal neurons and adult astrocytes. Nat Biotechnol.

[53] Liguore WA, Domire JS, Buttxon D, Wang Y, Dufour BD, Srinivasan S, et al. AAV-PHP.B administration results in a differential pattern of cns biodistribution in non-human primates compared with mice. Mol Ther. 2019; 27(11):2018-2037. 10.1016/j.ymthe.2019.07.017 PMC6838922 31420242

[54] Fu H, Dirosario J, Killedar S, Zaraspe K, McCarty DM (2011). Correction of neurological disease of mucopolysaccharidosis IIIB in adult mice by rAAV9 trans-blood-brain barrier gene delivery. Mol Ther.

[55] Foust KD, Salazar DL, Likhite S, Ferraiuolo L, Ditsworth D, Ilieva H (2013). Therapeutic AAV9-mediated suppression of mutant SOD1 slows disease progression and extends survival in models of inherited ALS. Mol Ther.

[56] Yamashita T, Chai HL, Teramoto S, Tsuji S, Shimazaki K, Muramatsu S (2013). Rescue of amyotrophic lateral sclerosis phenotype in a mouse model by intravenous AAV9-ADAR2 delivery to motor neurons. EMBO Mol Med.

[57] Dufour BD, Smith CA, Clark RL, Walker TR, McBride JL (2014). Intrajugular vein delivery of AAV9-RNAi prevents neuropathological changes and weight loss in Huntington's disease mice. Mol Ther.

[58] Varadi K, Michelfelder S, Korff T, Hecker M, Trepel M, Katus HA (2012). Novel random peptide libraries displayed on AAV serotype 9 for selection of endothelial cell-directed gene transfer vectors. Gene Ther.

[59] Smith RH, Levy JR, Kotin RM (2009). A simplified baculovirus-AAV expression vector system coupled with one-step affinity purification yields high-titer rAAV stocks from insect cells. Mol Ther.

[60] Wu Y, Jiang L, Geng H, Yang T, Han Z, He X (2018). A recombinant baculovirus efficiently generates recombinant adeno-associated virus vectors in cultured insect cells and larvae. Mol Ther Methods Clin Dev.

[61] Chen YH, Keiser MS, Davidson BL (2018). Adeno-associated virus production, purification, and titering. Curr Protoc Mouse Biol.

[62] Paxinos G, Franklin KBJ (2013). Paxinos and franklin’s the mouse brain in stereotaxic coordinates.

[63] Lau CH, Ho JW, Lo PK, Tin C (2019). Targeted transgene activation in the brain tissue by systemic delivery of engineered AAV1 expressing CRISPRa. Mol Ther Nucleic Acids.

[64] Bedbrook CN, Deverman BE, Gradinaru V. Viral strategies for targeting the central and peripheral nervous systems. In: Roska B, Zoghbi HY eds . Annual review of neuroscience, Vol 41. 2018. p. 323–348. 10.1146/annurev-neuro-080317-062048 29709207

[65] Kaplitt MG, Feigin A, Tang C, Fitzsimons HL, Mattis P, Lawlor PA (2007). Safety and tolerability of gene therapy with an adeno-associated virus (AAV) borne GAD gene for Parkinson's disease: an open label, phase I trial. Lancet.

[66] Peyvandi F, Garagiola I. Clinical advances in gene therapy updates on clinical trials of gene therapy in haemophilia. Haemophilia. 2019. 10.1111/hae.13816 31282050

[67] Yamaguti-Hayakawa GG, Ozelo MC (2019). Gene therapy: paving new roads in the treatment of hemophilia. Semin Thromb Hemost.

[68] McCarty DM, DiRosario J, Gulaid K, Muenzer J, Fu H (2009). Mannitol-facilitated CNS entry of rAAV2 vector significantly delayed the neurological disease progression in MPS IIIB mice. Gene Ther.

[69] Saleeba C, Dempsey B, Le S, Goodchild A, McMullan S (2019). A student's guide to neural circuit tracing. Front Neurosci.

[70] Li J, Liu T, Dong Y, Kondoh K, Lu Z (2019). Trans-synaptic neural circuit-tracing with neurotropic viruses. Neurosci Bull.

[71] Schwarz LA, Miyamichi K, Gao XJ, Beier KT, Weissbourd B, DeLoach KE (2015). Viral-genetic tracing of the input-output organization of a central noradrenaline circuit. Nature.

[72] Beier KT, Steinberg EE, DeLoach KE, Xie S, Miyamichi K, Schwarz L (2015). Circuit architecture of VTA dopamine neurons revealed by systematic input-output mapping. Cell.

[73] Bessis N, GarciaCozar FJ, Boissier MC (2004). Immune responses to gene therapy vectors: influence on vector function and effector mechanisms. Gene Ther.

[74] Kirik D, Rosenblad C, Burger C, Lundberg C, Johansen TE, Muzyczka N (2002). Parkinson-like neurodegeneration induced by targeted overexpression of alpha-synuclein in the nigrostriatal system. J Neurosci.

[75] Wu SH, Liao ZX, Zheng N, Zhang LH, Tang H (2017). Comparative study of the transfection efficiency of commonly used viral vectors in rhesus monkey (Macaca mulatta) brains. Zool Res..

[76] Towne C, Schneider BL, Kieran D, Redmond DE, Aebischer P (2010). Efficient transduction of non-human primate motor neurons after intramuscular delivery of recombinant AAV serotype 6. Gene Ther.

[77] Weiss AR, Liguore WA, Domire JS, Button D, McBride JL (2020). Intra-striatal AAV2.retro administration leads to extensive retrograde transport in the rhesus macaque brain: implications for disease modeling and therapeutic development. Sci Rep..

